# Acquired Immune Thrombotic Thrombocytopenic Purpura Following Transaxillary Transcatheter Aortic Valve Replacement: A Rare Life-Threatening Hematologic Emergency

**DOI:** 10.7759/cureus.116317

**Published:** 2026-09-15

**Authors:** Alexandre Tourmous, Nicolas Mubiayi Mujani, Anthony Dionso Diyabanza, Joelle Kefer

**Affiliations:** 1 Cardiology, Cliniques Universitaires Saint-Luc, Brussels, BEL

**Keywords:** adamts13 deficiency, plasma exchange, structural heart disease, tavr, thrombocytopenia, thrombotic microangiopathy, thrombotic thrombocytopenic purpura, transcatheter aortic valve replacement

## Abstract

Immune thrombotic thrombocytopenic purpura (iTTP) is a rare, life-threatening complication of transcatheter aortic valve replacement (TAVR). Because early thrombocytopenia after TAVR is common and usually benign, iTTP may be overlooked.

An 87-year-old man underwent successful transaxillary TAVR. His platelet count fell from 210 to 16×10³/µL within 72 hours, with hemoglobin declining from 9.5 to 6.8 g/dL. There were no bleeding, neurological, or renal manifestations. Laboratory findings were consistent with microangiopathic hemolytic anemia, and ADAMTS13 activity was <1% (French score 3), confirming iTTP. Treatment with plasma exchange, corticosteroids, caplacizumab, and rituximab led to rapid platelet recovery.

The diagnosis of iTTP should be considered in patients presenting with unexplained thrombocytopenia and hemolysis after TAVR, even when asymptomatic. Early recognition and prompt initiation of therapy are essential to improve outcomes.

## Introduction

Immune thrombotic thrombocytopenic purpura (iTTP) is a rare thrombotic microangiopathy characterized by severe ADAMTS13 deficiency, resulting in accumulation of ultra-large von Willebrand factor multimers and widespread platelet-rich microvascular thrombosis [1,2]. Historically fatal in more than 90% of cases, TTP still carries a mortality rate of 10% to 20% despite plasma exchange and immunosuppressive therapy [1,2]. The recent addition of caplacizumab to this regimen has further reduced early mortality, refractoriness, and exacerbations, with reported mortality rates below 5% in recent cohorts [2-4].

The estimated annual prevalence of TTP is approximately 10 cases per million people, with an annual incidence of about one new case per million [1]. The first acute episode occurs in adulthood in approximately 90% of cases, and the disease is mostly caused by an autoimmune mechanism, with inherited forms being exceptional [1]. TTP is about twice as frequent in women, and female sex, Black ethnicity, HLA-DRB1*11, and obesity have been identified as risk factors for the acquired form [1]. Older age is one of the factors associated with death and treatment refractoriness [1,2]. In this context, the occurrence of iTTP in an 87-year-old patient in the immediate aftermath of a structural cardiac intervention is exceptional.

Although acquired TTP has been reported after cardiac surgery, cardiopulmonary bypass, and vascular interventions, its occurrence following transcatheter aortic valve replacement (TAVR) remains extremely uncommon [5]. We report a rare case of severe acquired iTTP occurring shortly after transaxillary TAVR in an elderly patient who remained entirely asymptomatic despite profound hematological abnormalities.

## Case presentation

An 87-year-old man was referred for elective TAVR because of symptomatic severe degenerative aortic stenosis. His medical history included hypertension, chronic obstructive pulmonary disease (COPD) (Global Initiative for Chronic Obstructive Lung Disease (GOLD) grade II) with emphysematous changes, dyslipidemia, hypothyroidism, chronic kidney disease (CKD) stage IIIa according to Kidney Disease: Improving Global Outcomes (KDIGO) criteria, osteoporosis, microscopic colitis, and bilateral carotid artery stenosis. His chronic medications included low-dose aspirin, olmesartan, amlodipine, atorvastatin, levothyroxine, alendronate, pantoprazole, and alprazolam. No new drug had been introduced in the months preceding the procedure. He weighed 52 kg and was 165 cm tall (body mass index was 19.1 kg/m²). Comprehensive geriatric assessment identified him as frail, with a high risk of falls and of protein-energy malnutrition, and recommended post-procedural rehabilitation.

Preprocedural transthoracic echocardiography confirmed severe aortic stenosis, with an aortic valve area of 0.8 cm², a peak gradient of 92 mmHg and a mean gradient of 45 mmHg; left ventricular size and systolic function were preserved (ejection fraction 74%). N-terminal pro-B-type natriuretic peptide (NT-proBNP) was 427 pg/mL. Coronary angiography demonstrated single-vessel coronary artery disease (intermediate lesion of the mid circumflex artery). Computed tomography angiography showed an aortic annulus of 20.5 × 24.5 mm (area 398 mm², perimeter 72 mm), an aortic valve calcium score of 875, coronary ostia heights of 15 mm (left main) and 20 mm (right coronary artery), and sinus of Valsalva diameters of 29-30 mm; on the basis of these measurements, a 27 mm Navitor™ Vision self-expanding valve (Abbott Vascular, Santa Clara, USA) was selected. CT also revealed severe peripheral vascular disease and bilateral carotid stenosis (60-70% on the right carotid artery, 75-80% on the left carotid artery), rendering both transfemoral and transcarotid approaches unsuitable. A left transaxillary approach was therefore selected. The Society of Thoracic Surgeons (STS) predicted risk of mortality was 5.65%.

**Figure 1:**
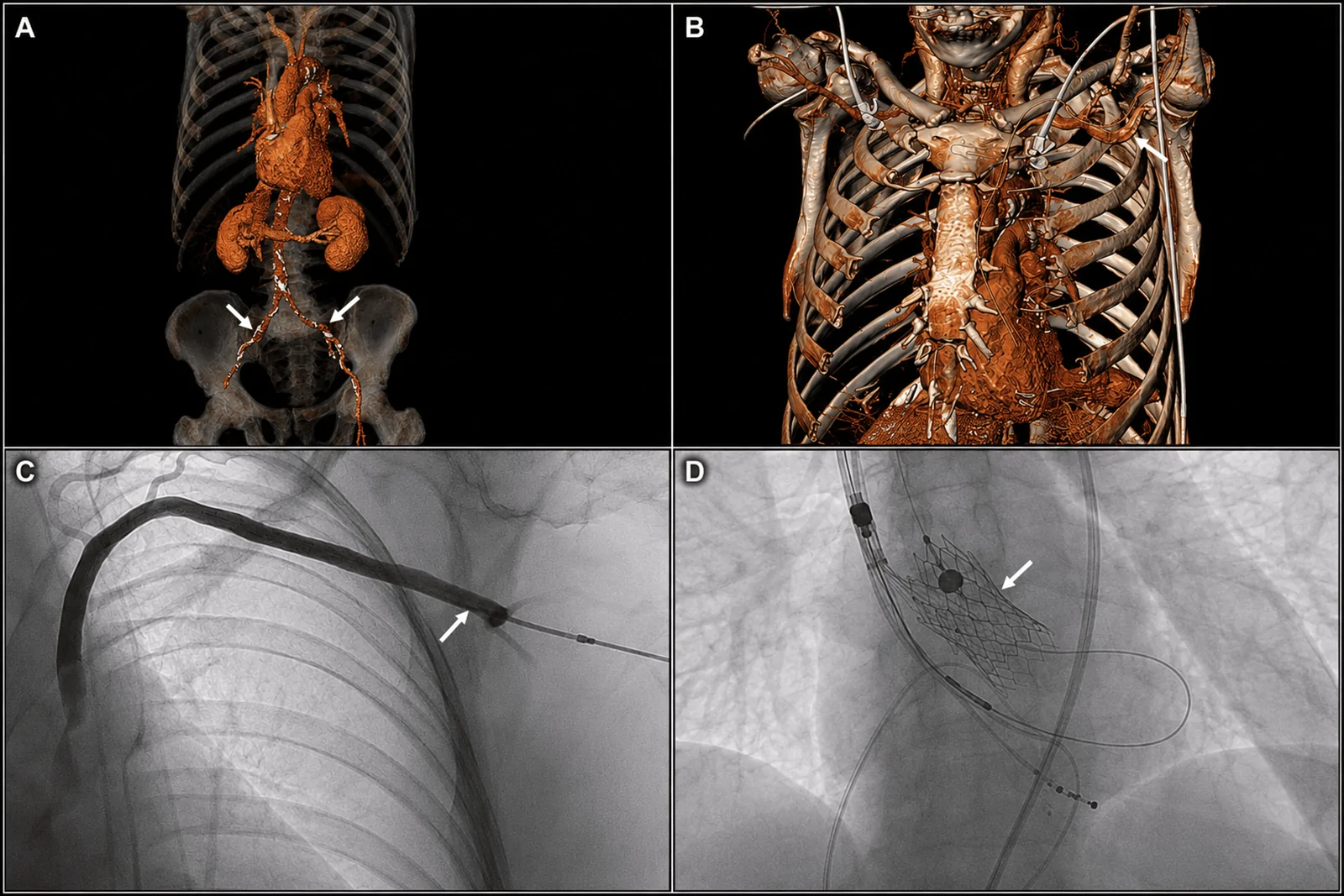
Anatomical evaluation and implantation of a transcatheter aortic valve through a left transaxillary approach. (A) Three-dimensional reconstruction of preprocedural computed tomography angiography demonstrating extensive vascular disease and unsuitable transfemoral access (arrows). (B) Three-dimensional reconstruction of preprocedural computed tomography angiography showing the selected left transaxillary approach (arrow). (C) Fluoroscopic image showing the left transaxillary access route (arrow) used for TAVR. (D) Final fluoroscopic view after implantation of the 27-mm Navitor^TM^ Vision aortic valve (Abbott Vascular, Santa Clara, USA) (arrow). TAVR: transcatheter aortic valve replacement

The patient underwent successful implantation of the valve following balloon valvuloplasty under rapid pacing. Persistent hemorrhage from the axillary access site occurred despite closure devices and required the implantation of an 8 × 60 mm Covera^TM^ Vascular Covered Stent (Bard Medical, Becton Dickinson, New Jersey, USA). Final angiographic results were satisfactory (Figure 1).

On the first postoperative day, hemoglobin decreased from 9.5 g/dL to 8.2 g/dL, which we initially attributed to the axillary hematoma, while platelet count remained at 152 ×10⁹/L. Transthoracic echocardiography and chest radiography showed no abnormalities, and the electrocardiogram showed sinus rhythm.

By postoperative day 2, hemoglobin had further declined to 7.4 g/dL and platelet count had declined to 58 ×10⁹/L. CT angiography excluded active bleeding and demonstrated a left axillary localized subcutaneous hematoma only. One unit of packed red blood cells was transfused.

**Figure 2:**
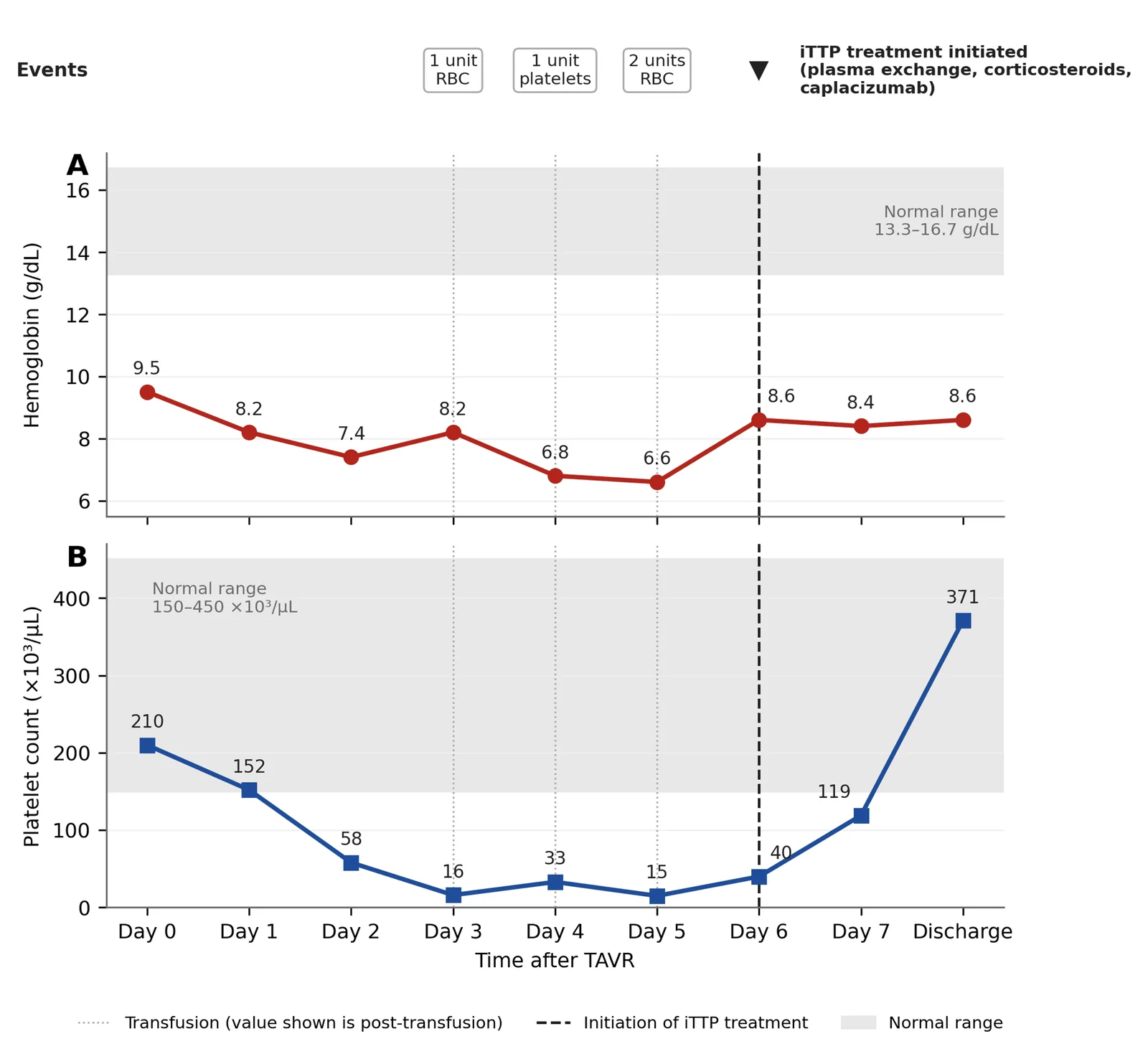
Evolution of hemoglobin (A) and platelet count (B) during hospitalization. Grey bands indicate normal ranges (hemoglobin 13.3-16.7 g/dL; platelets 150-450 ×10³/µL). Dotted lines indicate transfusions (values shown are post-transfusion); the dashed line indicates initiation of iTTP treatment (plasma exchange, corticosteroids, and caplacizumab) on day 6. RBC: red blood cells; TAVR: transcatheter aortic valve replacement; iTTP: immune thrombotic thrombocytopenic purpura

On postoperative day 3, platelet count reached a nadir of 16 ×10⁹/L and inflammatory markers increased significantly. Despite these abnormalities, the patient remained completely asymptomatic: he was afebrile, had no fluctuating neurological symptoms, and renal function remained stable (serum creatinine 1.42 mg/dL, compared with 1.39 mg/dL at baseline), so that only the two hematological components of the classic pentad (thrombocytopenia and microangiopathic hemolytic anemia) were present. By postoperative day 4, hemoglobin had decreased to 6.8 g/dL. The evolution of hemoglobin and platelet counts during hospitalization can be found in Figure 2. Given the clinical course, the hematology team was consulted for guidance in the management of the patient: laboratory investigations revealed elevated lactate dehydrogenase (LDH) (702 U/L), undetectable haptoglobin, and 1.4% schistocytes on peripheral blood smear, suggestive of microangiopathic hemolytic anemia; anti-PF4 antibodies were negative; ADAMTS13 activity was below 1%; and the French Score was 3, confirming acquired iTTP.

Surprisingly, the patient remained completely asymptomatic throughout the entire clinical course. Serial neurological examinations were normal (no confusion, focal neurological deficit, or seizure). The patient remained in sinus rhythm throughout hospitalization, and no myocardial ischemia, heart failure, gastrointestinal manifestations, or worsening renal dysfunction occurred.

Daily plasma exchange was initiated immediately together with corticosteroids (1 mg/kg), caplacizumab, and folic acid supplementation. Platelet counts recovered rapidly, exceeding 150 ×10⁹/L after two plasma exchange sessions. Plasma exchange was then discontinued, and rituximab was initiated under the hematology team's supervision. Regarding antithrombotic therapy, the patient was already on low-dose aspirin before TAVR, which was continued as single antiplatelet therapy after the procedure and maintained throughout the episode, including during severe thrombocytopenia; no P2Y12 inhibitor was added. At discharge, hemoglobin was 8.6 g/dL and platelet count 371 ×10⁹/L, and the patient was discharged on aspirin alone. Hematology follow-up was scheduled at discharge to ensure continued monitoring and treatment.

## Discussion

iTTP is a rare but life-threatening thrombotic microangiopathy resulting from severe ADAMTS13 deficiency [1,2]. The consequent accumulation of ultra-large von Willebrand factor multimers promotes spontaneous platelet aggregation and disseminated microvascular thrombosis, leading to thrombocytopenia, microangiopathic hemolytic anemia, and end-organ ischemia [1].

The present case is remarkable for several reasons. First, the temporal relationship between TAVR and the onset of iTTP strongly suggests a procedural trigger. To our knowledge, only one comparable case has been reported in the literature (China) [5]. Unlike the case reported by Shao et al. [5], which occurred after a complicated TAVR requiring emergency surgical valve replacement, our patient developed iTTP after a technically successful transaxillary procedure without surgical conversion. This observation suggests that transcatheter valve implantation itself may be sufficient to trigger the disease.

The pathophysiological mechanisms remain unclear. TAVR induces substantial endothelial activation and mechanical stress, leading to an increased release of von Willebrand factor multimers. In predisposed individuals, this intense endothelial stimulation may contribute to the development - or unmasking - of anti-ADAMTS13 autoantibodies. Furthermore, the hemorrhage requiring covered stent implantation, local hematoma formation, and inflammatory response may have represented additional triggering factors.

Another remarkable aspect of this case is the complete absence of symptoms despite severe biological abnormalities. Historically, TTP was described by the classical pentad of thrombocytopenia, hemolytic anemia, neurological manifestations, renal dysfunction, and fever [1,2]. However, fewer than 10% of patients present with the complete pentad [1]. Nevertheless, most patients exhibit at least some degree of neurological, cardiac, or renal involvement. In contrast, our patient remained entirely asymptomatic despite a platelet nadir of 16 ×10⁹/L and ADAMTS13 activity below 1%.

The diagnosis was initially challenging because anemia and thrombocytopenia are common after TAVR and may be attributed to bleeding, hemodilution, or platelet consumption (hypoattenuated leaflet thickening, or HALT). In our case, the presence of an access-site hematoma initially suggested hemorrhagic anemia. HALT refers to CT-detected thickening of bioprosthetic valve leaflets, considered a manifestation of subclinical valve thrombosis and potentially associated with reduced leaflet motion [6]. This etiology was ruled out in our case due to the absence of CT findings consistent with HALT. Hemolysis secondary to paravalvular leak was excluded, as echocardiography showed no evidence of paravalvular leakage. Drug-induced thrombotic microangiopathy was also considered; however, neither the patient's chronic medications nor the antiplatelet regimen prescribed after TAVR (aspirin alone, without thienopyridine) belongs to the drug classes classically implicated in TTP (such as ticlopidine, clopidogrel, quinine, calcineurin inhibitors, or chemotherapeutic agents), no new drug had been recently introduced, and the demonstration of severe ADAMTS13 deficiency pointed to an immune-mediated mechanism rather than a toxic one [1]. Ultimately, the disproportionate thrombocytopenia, schistocytosis, elevated LDH, undetectable haptoglobin, and severe ADAMTS13 deficiency established the diagnosis of iTTP.

Current international guidelines recommend immediate initiation of plasma exchange in all patients with suspected iTTP while awaiting ADAMTS13 results [7]. The addition of corticosteroids, rituximab, and caplacizumab has substantially improved outcomes and reduced disease-related mortality [3,4,7]. Our patient quickly received adequate therapy, resulting in rapid platelet recovery and complete hematological remission.

As TAVR continues to expand worldwide, clinicians should be aware of this rare but potentially fatal complication. Unexplained thrombocytopenia associated with hemolytic anemia after TAVR should prompt urgent evaluation for thrombotic microangiopathy, including peripheral blood smear examination and ADAMTS13 testing [1,5,7]. Early recognition and multidisciplinary management remain essential to prevent irreversible organ damage and death [1,7].

## Conclusions

Acquired iTTP is an exceptionally rare but potentially life-threatening complication following TAVR. This case highlights that iTTP may develop rapidly after the procedure and remain clinically silent despite profound hematological abnormalities. Therefore, unexplained or disproportionate thrombocytopenia associated with hemolytic anemia after TAVR should prompt early investigation for thrombotic microangiopathy, including peripheral blood smear and ADAMTS13 activity assessment. Prompt recognition and multidisciplinary management are crucial, as early initiation of targeted therapy can lead to rapid hematological recovery and prevent irreversible organ damage or death.
